# Regulation of Myofilament Contractile Function in Human Donor and Failing Hearts

**DOI:** 10.3389/fphys.2020.00468

**Published:** 2020-05-25

**Authors:** Kerry S. McDonald, Laurin M. Hanft, Joel C. Robinett, Maya Guglin, Kenneth S. Campbell

**Affiliations:** ^1^Department of Medical Pharmacology and Physiology, University of Missouri, Columbia, MO, United States; ^2^Division of Cardiovascular Medicine, University of Kentucky, Lexington, KY, United States; ^3^Department of Physiology, University of Kentucky, Lexington, KY, United States

**Keywords:** heart failure, human cardiac myocytes, contractile properties, rate of force development, loaded shortening, power output, sarcomere length

## Abstract

Heart failure (HF) often includes changes in myocardial contractile function. This study addressed the myofibrillar basis for contractile dysfunction in failing human myocardium. Regulation of contractile properties was measured in cardiac myocyte preparations isolated from frozen, left ventricular mid-wall biopsies of donor (*n* = 7) and failing human hearts (*n* = 8). Permeabilized cardiac myocyte preparations were attached between a force transducer and a position motor, and both the Ca^2+^ dependence and sarcomere length (SL) dependence of force, rate of force, loaded shortening, and power output were measured at 15 ± 1°C. The myocyte preparation size was similar between groups (donor: length 148 ± 10 μm, width 21 ± 2 μm, *n* = 13; HF: length 131 ± 9 μm, width 23 ± 1 μm, *n* = 16). The maximal Ca^2+^-activated isometric force was also similar between groups (donor: 47 ± 4 kN⋅m^–2^; HF: 44 ± 5 kN⋅m^–2^), which implicates that previously reported force declines in multi-cellular preparations reflect, at least in part, tissue remodeling. Maximal force development rates were also similar between groups (donor: *k*_*tr*_ = 0.60 ± 0.05 s^–1^; HF: k_*tr*_ = 0.55 ± 0.04 s^–1^), and both groups exhibited similar Ca^2+^ activation dependence of *k*_*tr*_ values. Human cardiac myocyte preparations exhibited a Ca^2+^ activation dependence of loaded shortening and power output. The peak power output normalized to isometric force (PNPO) decreased by ∼12% from maximal Ca^2+^ to half-maximal Ca^2+^ activations in both groups. Interestingly, the SL dependence of PNPO was diminished in failing myocyte preparations. During sub-maximal Ca^2+^ activation, a reduction in SL from ∼2.25 to ∼1.95 μm caused a ∼26% decline in PNPO in donor myocytes but only an ∼11% change in failing myocytes. These results suggest that altered length-dependent regulation of myofilament function impairs ventricular performance in failing human hearts.

## Introduction

Human heart failure is defined as the inability of the cardiac pump function to meet peripheral demands. Heart failure afflicts ∼6 million patients in the US and is associated with 1 in 9 deaths (Benjamin et al., 2018). Most current medication strategies for heart failure are designed to alleviate symptoms by decreasing the hemodynamic load (arterial blood pressure). Thus, new precision-based approaches that focus on the disease mechanisms intrinsic to the myocardium could have a significant impact.

The etiology of human heart failure is complex and encompasses ventricular remodeling and alterations to cardiac myocyte biology, which include energetic deficiencies and alterations in calcium handling and myofilament function. The purpose of this study was to address the myofibrillar basis for contractile dysfunction in failing human myocardium. Regulation of steady-state and dynamic contractile properties was measured in single permeabilized cardiac myocyte preparations isolated from frozen, left ventricular mid-wall biopsies of donor and failing human hearts.

## Materials and Methods

Samples were obtained from patients undergoing heart transplants at the University of Kentucky and from organ donors who did not have heart failure. Hearts were handed to a researcher as soon as these were excised from the body. Mid-wall myocardial samples of distal anterior left ventricular free wall were dissected and snap frozen in liquid nitrogen and stored at -150°C before shipping to the University of Missouri. All procedures were approved by the University of Kentucky Institutional Review Board, and written informed consent was obtained from each patient who had heart failure and from a legally authorized representative of each organ donor. Samples were obtained from seven organ donors (mean age of 39 ± 6, 3 female, 6 white, 1 African-American), four patients with ischemic heart failure (mean age of 48 ± 10, 1 female, 3 white, 1 African American), and four patients with non-ischemic heart failure (mean age of 51 ± 4, 3 female, 3 white, 1 African American). Data are presented as two groups, donor and heart failure (HF).

### Single Permeabilized Myocardial Samples and Functional Measurements

**FIGURE 1:**
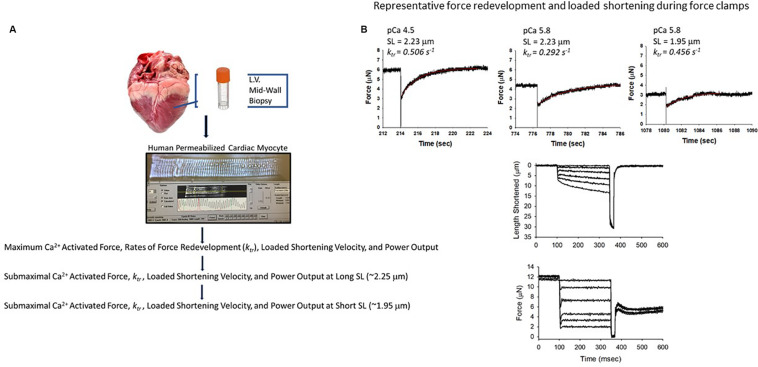
Experimental design. **(A)** Tissue was procured as part of the University of Kentucky Heart Transplant program and Biobank program. Mid-wall myocardial samples of distal anterior left ventricular free wall were dissected and snap frozen in liquid nitrogen and stored at -150°C before shipping to the University of Missouri. Single human permeabilized cardiac myocyte preparations were mounted between a force transducer and a motor, and contractile properties were measured during maximal and sub-maximal Ca^2+^ activations at long (∼2.25 μm) and short sarcomere lengths (∼1.95 μm). **(B)** Representative force redevelopment and loaded shortening traces from a human permeabilized cardiac myocyte preparation.

A total of 29 permeabilized single cardiac myocyte preparations from eight hearts from patients who had heart failure and seven hearts from organ donors were analyzed for this work (Table 1). Single permeabilized cardiac myocyte preparations (see Figure 1) were mounted between a force transducer and a motor, and contractile properties were measured during maximal and sub-maximal Ca^2+^ activations at long (∼2.25 μm) and short sarcomere lengths (∼1.95 μm), which are thought to span the sarcomere working range (Pollack and Huntsman, 1974; Grimm et al., 1980; Guccione et al., 1997; Chung et al., 2018). In brief, cardiac myocyte preparations were attached by placing the ends of the myocyte into stainless steel troughs (25 gauge) and ends were secured by overlaying a 0.5-mm length of 4–0 monofilament nylon suture (Ethicon, Inc.) and tying the suture into the troughs with loops of 10–0 monofilament (Ethicon, Inc.). The attachment procedure was performed under a stereomicroscope (∼100× magnification) using finely shaped forceps (McDonald, 2000). Prior to the mechanical measurements, the experimental apparatus was mounted on the stage of an inverted microscope (model IX-70, Olympus Instrument Co., Japan). Mechanical measurements were performed using a capacitance-gauge transducer plus a 10× amplifier (Aurora Scientific, Inc., Aurora, ON, Canada). Length changes were introduced using a DC torque motor (model 308, Aurora Scientific, Inc.) driven by voltage commands from a personal computer via a 16-bit D/A converter (AT-MIO-16E-1, National Instruments Corp., Austin, TX, United States). Force and length signals were digitized at 1 kHz and stored on a personal computer using LabVIEW for Windows (National Instruments Corp.). Sarcomere length was monitored using an IonOptix SarcLen system (IonOptix, Milton, MA, United States), which used a fast Fourier transform algorithm of the video image of the myocyte. Following attachment, the relaxed, permeabilized cardiac myocyte preparation was adjusted to a sarcomere length of ∼2.25 μm and passive tension was measured by slacking the preparation in pCa 9.0 solution (McDonald and Moss, 1995; McDonald, 2000). Cardiac myocyte preparation force, rates of force development, and force-velocity and power-load measurements were made at 15 ± 1°C as previously described (McDonald, 2000; Hinken and McDonald, 2004; Hanft et al., 2017).

**TABLE 1:** Human permeabilized cardiac myocyte preparations.

	Cardiac myocyte preparations	Length (μm)	Width (μm)	Sarcomere length (μm)	Passive force (kN⋅m^–2^)	Peak power (pW)	pCa for half-max. force	Relative force half-max. pCa
Donor	13	148 ± 10	21 ± 2	2.26 ± 0.01	1.91 ± 0.40	54 ± 12	5.77 ± 0.01	0.53 ± 0.03
HF	16	131 ± 9	23 ± 5	2.27 ± 0.01	1.67 ± 0.26	43 ± 25	5.85 ± 0.01	0.56 ± 0.02

*Values are means ± S.E.M.*

### Data and Statistical Analysis

Force redevelopment kinetics (*k*_*tr*_) following a slack-restretch maneuver were determined by fitting a single exponential function to the force recovery profile using the equation

F=F(1-e)-ktrtmax

where F is tension at time *t*, F_max_ is maximal tension, and *k*_*tr*_ is the rate constant of force development.

Myocyte length traces, force-velocity curves, and power-load curves were analyzed as previously described (McDonald, 2000). Myocyte length and sarcomere length traces during loaded shortening were fit to a single decaying exponential equation:

L=Ae+-⁣kt⁣C

where L is cell length at time *t*, A and C are constants with dimensions of length, and *k* is the rate constant of shortening (*k*_shortening_). Velocity of shortening at any given time, *t*, was determined as the slope of the tangent to the fitted curve at that time point. In this study, loaded shortening velocities were calculated at time equal to 100 ms after the onset of the force clamp.

Hyperbolic force-velocity curves were fit to the relative force-velocity data using the Hill equation (Hill, 1938)

(P+a)(V+b)=(P+oa)b

where P is force during shortening at velocity V, P_*o*_ is the maximal isometric force, and *a* and *b* are constants with dimensions of force and velocity, respectively. Force-velocity data were normalized to isometric force to illustrate condition effects on loaded shortening velocity. Power-load curves were obtained by multiplying force x velocity at each relative load on the force-velocity curve, and statistical analysis compared peak normalized power output (PNPO) values, which were obtained by multiplying relative force at optimum power x velocity at optimum power. Curve fitting was performed using a customized program written in QBasic, as well as commercial software (SigmaPlot).

The experimental data were analyzed in SAS 9.1.3 (SAS Institute, Cary, NC, United States) using linear mixed effects. As previously described (Haynes et al., 2014), this statistical approach accounts for the hierarchical structure of the data (values obtained from different numbers of myocyte preparations from hearts procured from organ donors and patients who had heart failure) and has greater statistical power than standard ANOVA for this type of design. Compound symmetry was assumed for the covariance structure, and *post-hoc* analyses were performed using Tukey-Kramer corrections. *P* < 0.05 was accepted as a statistically significant difference. *N* = number of hearts. *n* = number of cardiac myocyte preparations. Values are expressed as means ± SEM.

## Results

### Contractile Properties of Human Single Permeabilized Cardiac Myocyte Preparations

Permeabilized human cardiac myocyte preparations were similar in size between donor and heart failure groups (Table 1). At sarcomere length (SL) ∼2.25 μm, passive tension was similar between groups (donor: 1.91 ± 0.40 vs. HF: 1.67 ± 0.26 kN⋅m^–2^) (Table 1).

**FIGURE 2:**
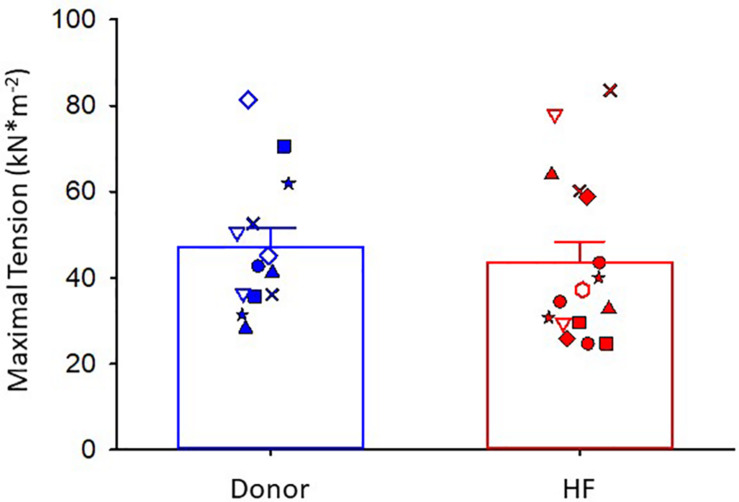
Maximal Ca^2+^-activated tension. Donor group, *N* = 7 hearts, *n* = 13 cardiac myocyte preparations. HF group, *N* = 8 hearts, *n* = 16 cardiac myocyte preparations. Different symbols indicate data points from each heart sample. Linear mixed model, *p* = 0.715.

Maximal Ca^2+^-activated tension was similar between donor and heart failure cardiac myocyte preparations (donor: 47 ± 4 kN⋅m^–2^; HF: 44 ± 5 kN⋅m^–2^) (Figure 2). For all figures, the different symbols indicate data points from each heart sample to indicate variability among tissues. After the entire experimental protocol (see Figure 1), the final maximal Ca^2+^-activated tension was 0.902 ± 0.037 of the initial pCa 4.5 force in donor preparations and 0.806 ± 0.032 in heart failure preparations (*p* = 0.064).

**FIGURE 3:**
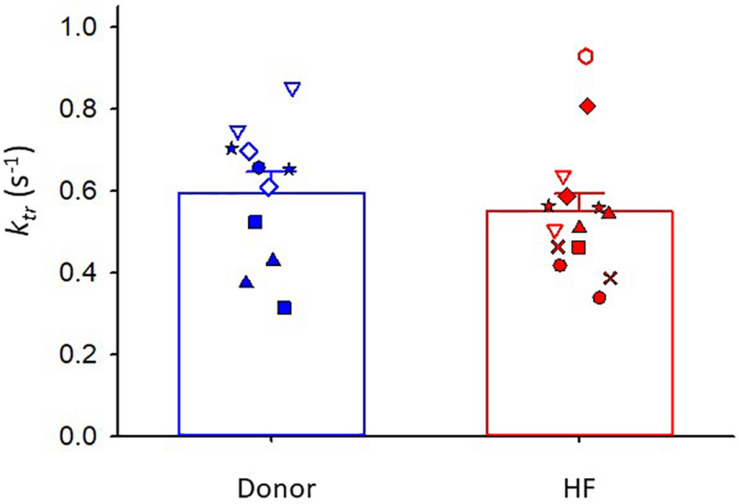
Rate of force development (*k*_*tr*_) during maximal Ca^2+^ activation. Donor group, *N* = 6 hearts, *n* = 11 cardiac myocyte preparations. HF group, *N* = 8 hearts, *n* = 14 cardiac myocyte preparations. Different symbols indicate data points from each heart sample. Linear mixed model, *p* = 0.663.

**FIGURE 4:**
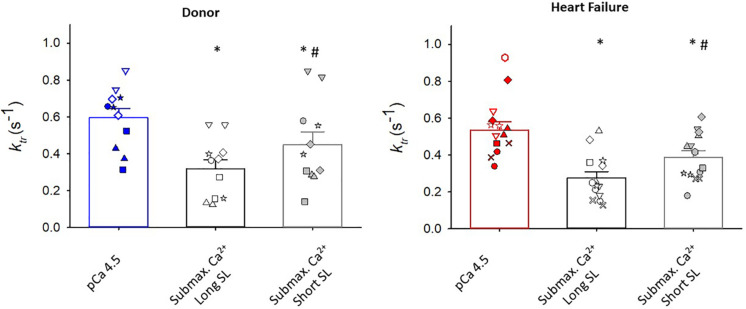
Regulation of rate of force development (*k*_*tr*_). In myocytes from organ donors (*N* = 6 hearts, *n* = 11 cardiac myocyte preparations) and patients with heart failure (*N* = 8 hearts, *n* = 14 cardiac myocyte preparations), *k*_*tr*_ values were lower during half maximal than maximal Ca^2+^ activations (*p < 0.001). At half-maximal Ca^2+^ activations, *k*_*tr*_ values were lower at long sarcomere length (^#^*p* < 0.001). Different symbols indicate data points from each heart sample. Linear mixed model testing for main effects of heart failure status, *k*_*tr*_ experimental condition, and their statistical interaction.

Maximal rates of force development (as indexed by the rate constant, *k*_*tr*_) were also similar between donor and heart failure groups Donor: *k*_*tr*_ = 0.60 ± 0.05 s^–1^; HF: k_*tr*_ = 0.55 ± 0.04 s^–1^) (Figure 3). All human cardiac myocyte preparations exhibited a robust Ca^2+^ activation dependence of *k*_*tr*_, i.e., lower *k*_*tr*_ values at half-maximal Ca^2+^ activation (*p* < 0.001) (Figure 4). In addition, *k*_*tr*_ values increased at short sarcomere length (SL) compared with those at long SL during sub-maximal Ca^2+^ activations (*p* < 0.001).

**FIGURE 5:**
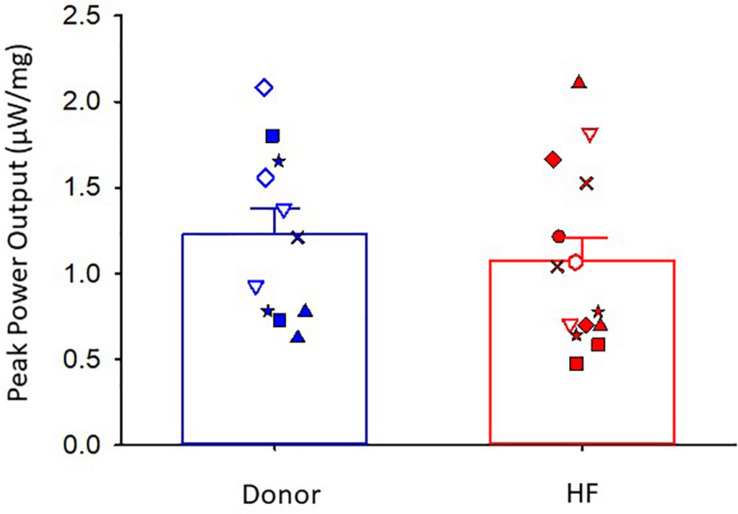
Peak absolute power output. Donor group, *N* = 6 hearts, *n* = 11 cardiac myocyte preparations. HF group, *N* = 8 hearts, *n* = 14 cardiac myocyte preparations. Different symbols indicate data points from each heart sample. Linear mixed model, *p* = 0.542.

**FIGURE 6:**
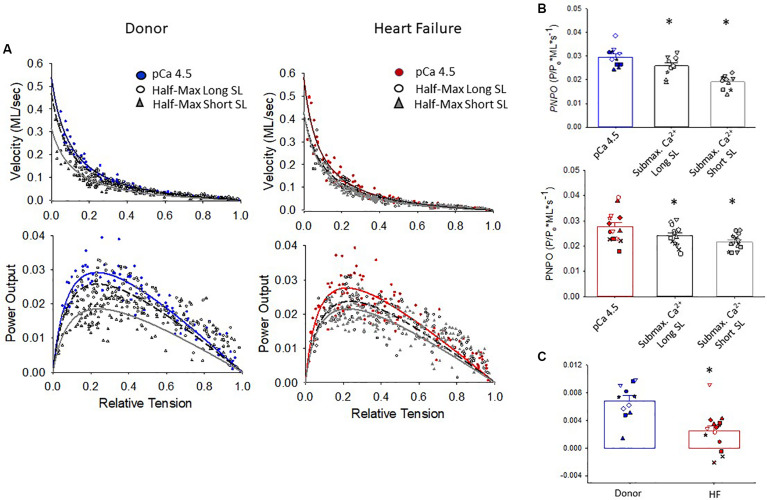
Normalized force-velocity and power-load curves. **(A)** Cumulative force-velocity and power-load curves from donor and HF preparations. **(B)** In myocyte preparations from organ donors and patients with heart failure, the peak normalized power output (PNPO) was lower at half-maximal Ca^2+^ activation than at maximal Ca^2+^ activation (*p = 0.027 or lower depending on comparisons). **(C)** Sarcomere length dependence of PNPO (that is, PNPO at long SL half max – short SL half-max) was significantly reduced in failing samples, *p = 0.004. Different symbols indicate data points from each heart sample. All tests were performed using the linear mixed model.

The maximal absolute power generating capacity was similar between donor and heart failure groups (donor: 1.23 ± 0.15 μW⋅mg^–1^; HF: 1.07 ± 0.14 μW⋅mg^–1^) (Figure 5). Statistical analysis showed that the peak normalized power output (PNPO) fell as Ca^2+^ activation was reduced in cells from organ donors and patients who had heart failure (Figures 6A,B). At sub-maximal Ca^2+^ activations, PNPO also decreased at short sarcomere length in both groups. Interestingly, Figure 6C shows that cells from failing hearts exhibited a markedly reduced sarcomere length dependence of PNPO (*p* = 0.004). This indicates that normalized power falls further as sarcomere length is reduced in cells from organ donors than it does in cells from patients who had heart failure.

No differences were observed in either maximal Ca^2+^-activated tension or *k*_*tr*_ values between sexes in cardiac myocyte preparations from donor or heart failure samples (Supplementary Figures S1, S2).

## Discussion

### Brief Summary

Steady-state and dynamic contractile properties were measured in human single permeabilized cardiac myocyte preparations. Human cardiac myocytes exhibited both Ca^2+^ activation and sarcomere length dependence of force development and power output. Maximal Ca^2+^-activated contractile force, rates of force development, and power were similar between human donor and failing samples. Conversely, the sarcomere length dependence of power was diminished in myofilaments from failing hearts, which provides a sub-cellular, biophysical mechanism for the depressed Frank-Starling relationship in failing human hearts.

### Human Permeabilized Cardiac Myocyte Preparations

For both donor and heart failure samples, human single permeabilized cardiac myocyte preparations displayed Ca^2+^ activation dependence of force, *k*_*tr*_ values, loaded shortening, and power output, which has previously been reported in rodent and porcine permeabilized cardiac myocyte preparations (McDonald, 2000; McDonald et al., 2012). Previous studies have reported the Ca^2+^ sensitivity of both force (Wolff et al., 1996; van der Velden et al., 2000, van Der Velden et al., 2001; van der Velden et al., 2003, 2006; Kooij et al., 2010; Wijnker et al., 2013; Haynes et al., 2014; Swenson et al., 2017) and rates of force development (Edes et al., 2007) in human permeabilized cardiac myocyte preparations. Our study is the first, to our knowledge, to show the Ca^2+^ activation dependence of loaded shortening and power output in human permeabilized cardiac myocyte preparations. Compared with those during maximal Ca^2+^ activations, the velocity at optimal power, peak normalized power output, and absolute power output were all lower during half-maximal Ca^2+^ activations, which signifies that changes in both force and loaded shortening velocity account for the regulation of human myofilament power output generating capacity by activator [Ca^2+^]. Also, human cardiac myocyte preparations exhibited a sarcomere length dependence of force, *k*_*tr*_ values, loaded shortening, and power output, similar to reports on rodent (Fabiato and Fabiato, 1975; Adhikari et al., 2004; Stelzer and Moss, 2006; Korte and McDonald, 2007; Hanft and McDonald, 2009, 2010; Hanft et al., 2016) and porcine (McDonald et al., 2012) permeabilized cardiac myocyte preparations.

### Donor vs. Heart Failure Human Permeabilized Cardiac Myocyte Preparations

Maximal Ca^2+^-activated tension, *k*_*tr*_ values, and absolute power output were similar between donor and heart failure cardiac myocyte preparations. Other studies have reported similar maximal Ca^2+^-activated tensions in human single permeabilized cardiac myocytes obtained from donor and end-stage heart failure samples stored in liquid nitrogen (Wolff et al., 1996; van der Velden et al., 2000, van Der Velden et al., 2001; van der Velden et al., 2003, 2006). In contrast, maximal Ca^2+^-activated tension has been reported to be reduced in single permeabilized cardiac myocytes from small-animal mixed models of heart failure (Belin et al., 2006; de Waard et al., 2007; Edes et al., 2008; Hanft et al., 2017). In addition, *k*_*tr*_ values, loaded shortening, and power output have been reported to be decreased in single permeabilized myocytes from late-stage heart failure in rodent models (Edes et al., 2008; Hanft et al., 2017). The exact reasons for the differences between human and rodent heart failure studies are uncertain but likely arise, at least in part, from confounding variables associated with human patients (e.g., age, sex, medications, disease etiology, genetic variability, fresh vs. frozen samples). Consistent with this, contractile properties showed more variability in human cardiac myocyte preparation in this study compared with those in previous reports on rodent permeabilized cardiac myocyte preparations (Edes et al., 2008; Hanft et al., 2017).

### Length-Dependent Activation of Power in Human Permeabilized Cardiac Myocyte Preparations From Donor vs. Heart Failure Samples

The relationship between ventricular filling and ventricular output was described in the early twentieth century and is known as the Frank-Starling relationship (Frank, 1895; Patterson and Starling, 1914). The functional importance of the Frank-Starling relationship is to dynamically match left and right ventricular outputs and adapt hemodynamics to peripheral demands. The molecular mechanism underlying the Frank-Starling relationship involve sarcomere physical factors, including alterations in inter-filament lattice spacing (Fuchs and Martyn, 2005) and myosin cross-bridge orientation (Ait Mou et al., 2016), perhaps mediated by titin (Cazorla et al., 2001; Fukuda et al., 2003). The Frank-Starling relationship often is markedly depressed in late-stage cardiac failure (Braunwald and Ross, 1964; Ross and Braunwald, 1964; Holubarsch et al., 1996). Depressed Frank-Starling relationships result, at least in part, from the reduced length dependence of the activation of cardiac myofilaments from failing hearts. For instance, studies have reported reduced the length dependence of the Ca^2+^ sensitivity of force in single permeabilized cardiac myocyte preparations from failing human hearts (Schwinger et al., 1994; Holubarsch et al., 1996; Sequiera et al., 2013; Wijnker et al., 2014). Our study extended previous studies by examining the length dependence of cardiac myofilament loaded shortening and power output, which ultimately determines ventricular ejection. Interestingly, the length dependence of power was significantly depressed in failing human cardiac myocyte preparations, providing evidence of novel dynamic myofibrillar mechanisms (in addition to the length dependence of steady-state force) for depressed Frank-Starling relationships in failing human hearts. The exact molecular mechanisms for the depressed myofilament length dependence of power remain to be determined. Certainly, lower levels of PKA-mediated phosphorylation of cardiac myofilament proteins with heart failure may contribute (Wolff et al., 1996; van der Velden et al., 2003; Messer et al., 2007; Copeland et al., 2010; Kooij et al., 2010; Haynes et al., 2014). For instance, PKA phosphorylation and troponin exchange with pseudo-phosphorylation affect the length dependence of force (Kooij et al., 2010; Wijnker et al., 2013, 2014) and have been shown to, in part, restore the length dependence of the Ca^2+^ sensitivity of force in failing cardiac myocyte preparations. Another plausible molecular regulator of the length dependence of power is cardiac myocyte binding protein-C (cMyBP-C), a protein that resides in the C-zone of thick filaments and the phosphorylation state is depressed in failing myocardium (Hamdani et al., 2008; Copeland et al., 2010; Kooij et al., 2010; Haynes et al., 2014; Stathopoulou et al., 2016). In addition, preliminary studies from our lab have shown that the sarcomere length dependence of power is regulated by PKA phosphorylation sites on cMyBP-C in a transgenic mouse (Hanft et al., 2019). This finding raises the intriguing possibility that thick filament ON state may contribute to the length dependence of power output, which could be addressed experimental by small molecules or peptides that regulate thick filament states (Green et al., 2016). There also may be many dynamic changes in sarcomere signals beyond PKA, and the dynamic signaling at the sarcomere may vary widely between “normal” and heart failure myocardia, which requires exploration of sarcomere functional proteomics in response to varied stimuli, e.g., neurohumoral factors, activation frequency (i.e., heart rate), and medications (Hamdani et al., 2008).

### Integration of Findings in Failing Human Myocardium

Heart failure is postulated to arise from a variety of changes in cardiac myocyte biology, including loss of cells, morphological changes, transformed intracellular signaling, disrupted energetics, altered Ca^2+^ handling, and modifications of myofilament function. There is evidence of several contractility adaptations of intact myocardial preparations from failing human hearts, which include (*i*) decreased β-adrenergic responsiveness, (*ii*) depressed force-frequency relations, and (*iii*) slowed relaxation rates (Chung et al., 2018). From a myofilament standpoint, findings from failing permeabilized myocardial preparations include the (*i*) increased Ca^2+^ sensitivity of force, (*ii*) depressed length dependence of the Ca^2+^ sensitivity of force, and (*iii*) decreased PKA phosphorylation of myofilament proteins. One parameter that has yielded mixed results, especially between permeabilized multi-cellular and single-cell preparations, is maximal Ca^2+^-activated tension. In this regard, a recent study showed depressed force and power in multi-cellular preparations obtained from the transmural region of the left ventricular free wall of human failing myocardium (Haynes et al., 2014). Our current study directly addressed the sub-cellular mechanistic underpinnings of this finding by measuring force and power in single permeabilized cardiac myocytes obtained from the same region of the left ventricles of donor and failing myocardia. Our finding that force and power in single cells does not depend on the heart failure status implies that the changes in multi-cellular myocardial preparations arise, at least in part, from tissue remodeling, perhaps due to the loss of myofilaments and consequent augmentation of myocardial fibrosis (Haynes et al., 2014).

### Study Limitations

There are several limitations with human cardiac myocyte contractile property studies. First, there is the caveat of categorizing non-failing, donor myocardium as normal because of the possibility of high blood levels of catecholamines and inotropic support at the time of procurement. Second, the diversity of factors that contribute to non-ischemic disease likely augments experimental variability. Third, any assessment of cardiac myocyte size is precluded because ventricular biopsies are mechanically disrupted, which yields highly variable sized fragments. Fourth, permeabilized cardiac myocyte contractile property studies have an inherent bias due to the selection of rod-shaped cardiac myocyte-sized preparations. The selection of myocyte preparations (to only those that can withstand measurements) may exclude populations with, for instance, diminished maximum tension-generating capacity. The preparation selection process increases the possibility of a false-negative result about tension generation capacity. Fifth, the labor-intensive nature of single cardiac myocyte contractile property measurements limits the sample size, which in combination with regional variability (Kuro-o et al., 1986; Cazorla et al., 2000), disease etiology, and varied treatments also increases the likelihood of false-negative results. Sixth, the small size of single cardiac myocyte preparations (tens of nanograms) tests the limits of conventional techniques to assess myofilament protein isoforms and post-translational modifications in preparations that had their mechanics measured (Herron et al., 2001; Herron and McDonald, 2002; Korte et al., 2005; Hanft et al., 2013).

### Next Steps

This study provides a comprehensive assessment of contractile properties of human single permeabilized cardiac myocytes from donor and patients who have heart failure. The study implicates myocardial tissue remodeling to explain, at least in part, previous reports of depressed force and mechanics in human multi-cellular myocardial preparations. Additionally, since the sarcomere length dependence of power was diminished in myofilaments from failing hearts, it is likely that that altered length dependence of myofilament power contributes to the depressed Frank-Starling relationship in failing human hearts. Future exploration of unique molecular mechanisms will necessitate next-generation, unbiased, cellular functional proteomic approaches on preparations that had their mechanics measured. Additional work is needed to address other myofilament properties, including dynamic response to calcium transients, myofilament efficiency, thin and thick filament dynamics, and relaxation kinetics, and how these are affected during the progression to heart failure. Future investigations will also require systematic delineation of how confounders such as genetics, disease etiology, environment, age, sex, co-morbidities, and medications affect myofilament function studies. Both the expansion of dynamic myofilament studies and critical evaluation of individual patient scenarios are necessary for future, best-practice precision medicine to treat the underlying molecular mechanisms that cause human hearts to fail.

## Data Availability Statement

The datasets generated for this study are available on request to the corresponding author.

## Ethics Statement

All procedures were approved by the University of Kentucky Institutional Review Board and written informed consent was obtained from each patient who had heart failure and from a legally authorized representative of each organ donor, subjects gave informed consent.

## Author Contributions

KM, LH, and JR performed human single cardiac myocyte preparation experiments. MG and KC procured human tissue. KM, LH, JR, MG, and KC contributed to data analysis and manuscript preparation.

## Conflict of Interest

The authors declare that the research was conducted in the absence of any commercial or financial relationships that could be construed as a potential conflict of interest.

## Supplementary Material

FIGURE S1 Maximal Ca^2+^ activated tension- Effects of sex. Male, *N* = 8 hearts, *n* = 15 cardiac myocyte preparations. Female, *N* = 7 hearts, *n* = 14 cardiac myocyte preparations. Different symbols indicate data points from each heart sample. Linear mixed model, *p* = 0.549.

FIGURE S2 Rate of force development (*k*_*tr*_) during maximal Ca^2+^ activation- Effects of sex. Male, *N* = 7 hearts, *n* = 11 cardiac myocyte preparations. Female, *N* = 7 hearts, *n* = 12 cardiac myocyte preparations. Different symbols indicate data points from each heart sample. Linear mixed model, *p* = 0.993.
