# Supplementary figures and images for: Regulation of Myofilament Contractile Function in Human Donor and Failing Hearts
Source: Front Physiol. 2020 May 25;11:468. doi: 10.3389/fphys.2020.00468 (PMC7261867; doi:10.3389/fphys.2020.00468)

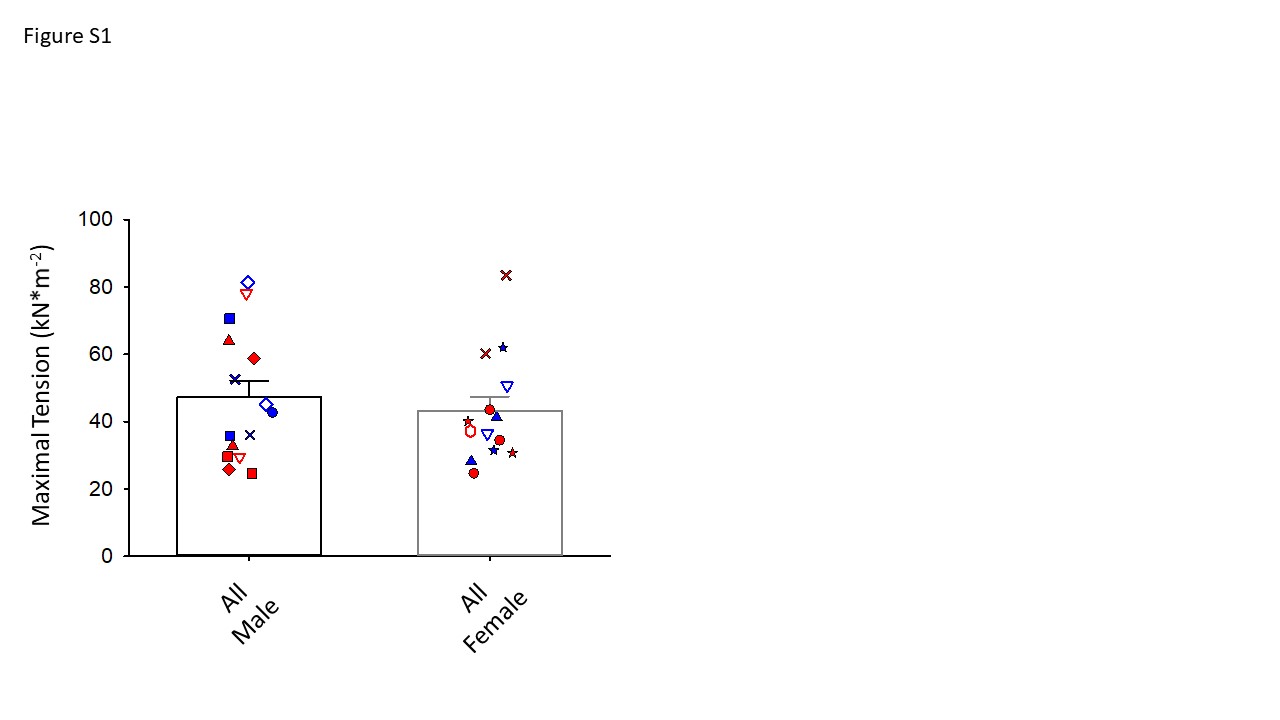

Supplement: FIGURE S1 — Maximal Ca2+ activated tension- Effects of sex. Male, N = 8 hearts, n = 15 cardiac myocyte preparations. Female, N = 7 hearts, n = 14 cardiac myocyte preparations. Different symbols indicate data points from each heart sample. Linear mixed model, p = 0.549. [file Image_1.JPEG]

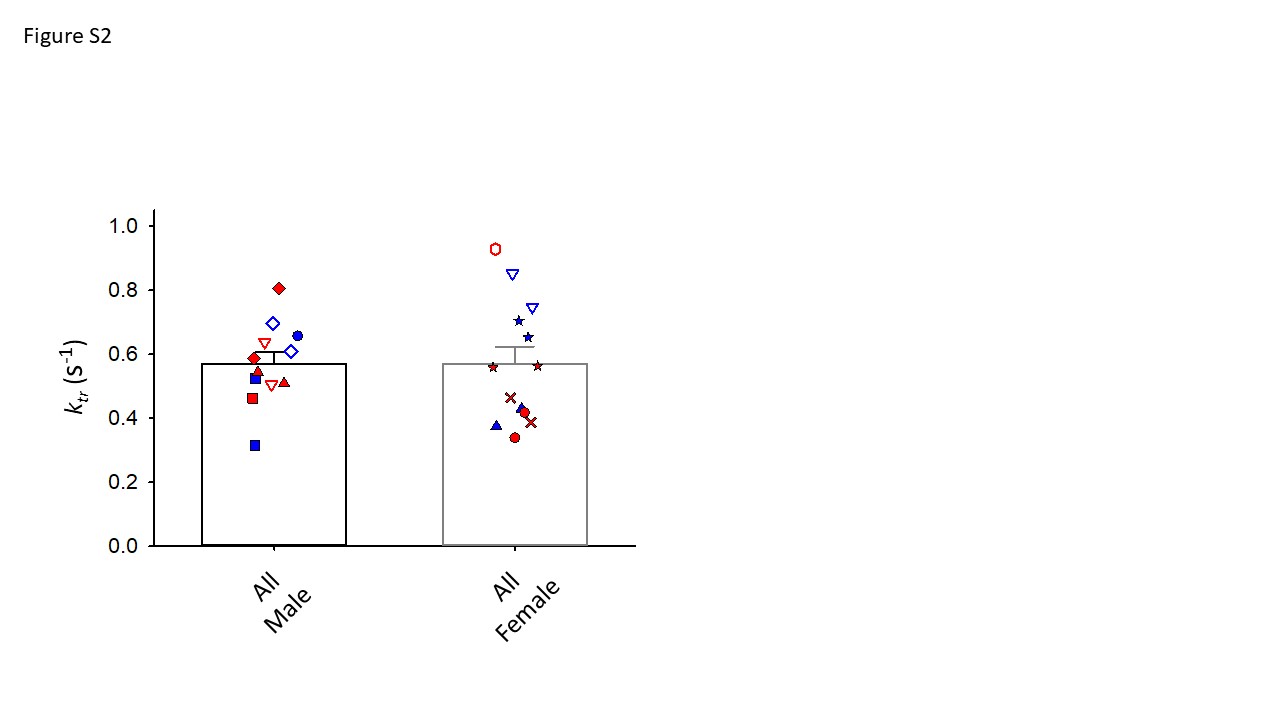

Supplement: FIGURE S2 — Rate of force development (ktr) during maximal Ca2+ activation- Effects of sex. Male, N = 7 hearts, n = 11 cardiac myocyte preparations. Female, N = 7 hearts, n = 12 cardiac myocyte preparations. Different symbols indicate data points from each heart sample. Linear mixed model, p = 0.993. [file Image_2.JPEG]
